# Analyses of Hypomethylated Oil Palm Gene Space

**DOI:** 10.1371/journal.pone.0086728

**Published:** 2014-01-30

**Authors:** Eng-Ti L. Low, Rozana Rosli, Nagappan Jayanthi, Ab Halim Mohd-Amin, Norazah Azizi, Kuang-Lim Chan, Nauman J. Maqbool, Paul Maclean, Rudi Brauning, Alan McCulloch, Roger Moraga, Meilina Ong-Abdullah, Rajinder Singh

**Affiliations:** 1 Advanced Biotechnology and Breeding Centre, Malaysian Palm Oil Board, Kajang, Selangor, Malaysia; 2 AgResearch Ruakura Research Centre, Hamilton, New Zealand; 3 AgResearch Invermay Agricultural Centre, Mosgiel, New Zealand; 4 AgResearch Grasslands Research Centre, Palmerston North, New Zealand; Temasek Life Sciences Laboratory, Singapore

## Abstract

Demand for palm oil has been increasing by an average of ∼8% the past decade and currently accounts for about 59% of the world's vegetable oil market. This drives the need to increase palm oil production. Nevertheless, due to the increasing need for sustainable production, it is imperative to increase productivity rather than the area cultivated. Studies on the oil palm genome are essential to help identify genes or markers that are associated with important processes or traits, such as flowering, yield and disease resistance. To achieve this, 294,115 and 150,744 sequences from the hypomethylated or gene-rich regions of *Elaeis guineensis* and *E. oleifera* genome were sequenced and assembled into contigs. An additional 16,427 shot-gun sequences and 176 bacterial artificial chromosomes (BAC) were also generated to check the quality of libraries constructed. Comparison of these sequences revealed that although the methylation-filtered libraries were sequenced at low coverage, they still tagged at least 66% of the RefSeq supported genes in the BAC and had a filtration power of at least 2.0. A total 33,752 microsatellites and 40,820 high-quality single nucleotide polymorphism (SNP) markers were identified. These represent the most comprehensive collection of microsatellites and SNPs to date and would be an important resource for genetic mapping and association studies. The gene models predicted from the assembled contigs were mined for genes of interest, and 242, 65 and 14 oil palm transcription factors, resistance genes and miRNAs were identified respectively. Examples of the transcriptional factors tagged include those associated with floral development and tissue culture, such as homeodomain proteins, MADS, Squamosa and Apetala2. The *E. guineensis* and *E. oleifera* hypomethylated sequences provide an important resource to understand the molecular mechanisms associated with important agronomic traits in oil palm.

## Introduction

The oil palm is a perennial crop that belongs to the family Arecaceae and the genus Elaeis [1]. There are two species in the genus - *Elaeis guineensis* (EG), the African oil palm and *Elaeis oleifera* (EO), of American origin [2]. EG is widely grown in the humid tropics (South-East Asia, Equatorial America, Africa and South Pacific) [3], and has become one of the most important crop in Malaysia and Indonesia. In order to remain competitive with other vegetable oil crops, there is a need to boost its yield and improve oil quality, for both of which deciphering its genome is key – to better understand the complexity of gene expression and interactions. One of the methods used is to identify genes expressed in a tissue of interest. The expressed sequence tag (EST) approach coupled with conventional sanger sequencing [4] was initially used to obtain information on gene diversity and mRNA expression patterns from various oil palm tissues [5]–[7]. However, the method is limited in utility, mostly identifying the abundantly expressed genes [8]. Although alternatives, such as constructing normalized cDNA libraries [9] have been tried, the method was deemed technically demanding.

The development of next generation sequencing (NGS) resolved these issues and identification of low abundance genes was thus made possible [10]. In oil palm, NGS sequencing was able to provide an in depth view of the genes expressed in flowers and fruit development. Comparing flowers of normal and abnormal clonal palms, Shearman and colleagues [11] identified a large number of differentially expressed genes, including those involved in chromatin remodelling and histone methylation. The abnormal palms in the study produced mantled fruits, a form of abnormality observed in palms produced via somatic embryogenesis. Shearman and colleagues [11] results are encouraging as previous studies have linked the occurrence of mantled fruits to changes in methylation [12]–[14]. In fruit development, Bourgis et al. [15] and Tranbarger et al. [16] were able to determine the expression of a new oil palm WRINKLED1 (WRI1) homolog, known to be involved in fatty acid biosynthesis in other plants. The expression of the gene correlated with those of several fatty acid biosynthetic genes in the mesocarp of oil palm. Nevertheless, the master regulator of WRI1 remains elusive [15], [16]. In both cases, access to the whole complement of genes – which can only be achieved by whole genome sequencing would enable hypothesis-driven experiments to be carried out and allow further investigations.

However, whole genome sequencing for complex organisms is costly and requires specialized expertise to navigate the data. This is more so for oil palm, with a genome of ∼1,800 Mb [17] is much larger than most oil seed crops [18]–[21] and model crops, such as rice (420 to 466 Mb) [22], [23] and *Arabidopsis thaliana* (∼135 Mb) [24]. However, the availability of NGS technology has recently allowed the sequencing of the oil palm genome [25]. Nevertheless, generating genomic sequence information through methylation filtration, a technique that allows for the preferential selection of hypomethylated regions of the genome [26], [27] and Sanger technology provides a comprehensive view of the genic regions of the genome. The method is based on chemical discrimination of repeated DNA from genes by certain strains of bacteria [27] resulting in the generation of a comprehensive gene coverage without the need for whole genome sequencing. The GeneThresher® (GT) methylation filtration technique has been validated in over a dozen plant genomes spanning all the major branches of the plant kingdom. It has been employed to generate comprehensive gene sets in ryegrass, clover, corn [28] and sorghum [29], where in sorghum, up to 96% of the genes were successfully tagged. The GT sequences were also an important source of microsatellite markers for application in genetic diversity [30] and genetic mapping [31] research programmes. This study reports on the sequencing and characterization of the hypomethylated regions of the oil palm genome, which is an important resource that focuses on the active regions of the genome.

## Materials and Methods

### Genomic library construction and methylation filtering

Nuclear genomic DNA was purified from the spear leaf of 7 EG and 2 EO palms, randomly sheared and size selected (0.6 to 1.4 kb). The fragments were ligated to a plasmid vector and transformed into DH5a (methylation filtering strain) or DH10b (non-methylation filtering strain) to generate GT (filtered) or whole genome (UF, unfiltered) libraries, respectively. UF libraries were used as negative control to determine the efficacy of filtered GT libraries. Nine filtered and nine unfiltered libraries were constructed (Table S1). The transformed strains were plated, DNA isolated from colonies, and end-sequenced with 3730 sequencing technology (Life Technologies Corp). For the oil palm bacterial artificial chromosomes (BAC), high molecular weight nuclear DNA was purified from an EG palm, embedded in agarose plugs, partially digested with *Hin*dIII, size selected and cloned into the CopyControl™ pCC1BAC™ (*Hin*dIII Cloning-Ready) Vector. DNA from individual BAC was prepared and four equimolar pools were constructed (∼44 BAC/pool) representing ∼10 megabases of the oil palm genome. Paired-end libraries were constructed from a 3–4 kb fraction of randomly sheared pooled BAC DNA using Roche 454 titanium kits, and sequenced to ∼30 fold coverage using Roche 454 XL sequencing technology.

### Sequence assembly

A graph-based clustering algorithm (MCL) and CAP3 [32] assembler were used for assembly. Graph based sequence clustering uses a data structure in which each sequence is a node of a graph, and each edge is a weighted connection between sequences. When clustering paired-end sequence data, edges are entered both to indicate sequence similarity, and mate-pairs, with the weighting based on alignment score for similar sequences, and a nominal weighting for mate-pairs. An initial own-versus-own BLAST [33] of sequences was performed, using an e-value cut-off of 1e^−10^ to ensure that the sequences that overlap by ≥40 base pairs (the minimum to join a contig), should report a BLAST hit. In the initial own-versus-own run, only the best hit of each sequence (apart from itself) was reported, so that each sequence only formed a link to at most one other sequence in the graph. The MCL algorithm was then executed to form clusters, which were then assembled into first-pass contigs. In addition, most singletons were identified in this phase, and excluded from further processing. The first pass analysis was designed to remove most of the redundancy in the data. A second-pass analysis was then initiated, in which an own-versus-own BLAST of the first-pass contigs against themselves was executed to report all hits. This forms the complete graph with each first-pass contig having outgoing graph links to all other first-pass contigs it hits, and clusters were formed again. These clusters-of-first-pass-contigs were then used to partition the original sequences into final bins of sequences. The first-pass contigs were discarded once the second pass clustering was completed. The final bins of original sequences were assembled using CAP3.

### Filter Power

Filter power (FP), which is the ratio of the probability that a filtered read sampled a gene coding sequence over the probability that an unfiltered read sampled a gene coding sequence was calculated according to Bedell et al. [29] (Materials S1). The estimation of FP was validated using a second method based on the statistics of the sequence assembly, following Whitelaw et al. [28], where the number of islands observed in the filtered and unfiltered assemblies were used to infer the effective sizes of the genomes sampled, using the formula of Lander and Waterman [34]. Details of the adaptation of the Lander Waterman formula and of related calculations are described in Materials S2. The size of the genome sampled was estimated by dividing the oil palm genome size (∼1,800 Mb for both species) by FP.

### Gene models and protein translations

*Ab initio* gene models were predicted using Augustus [35], SNAP [36] and GeneMark [37] implemented in MAKER [38]. Models trained on maize and rice data were used in Augustus and SNAP respectively, as they were the only monocot models in those programs. GeneMark, on the other hand, was trained on data from the oil palm BAC contigs. As MAKER carries out evidence based gene model predictions, sequences from the Swiss-Prot protein database were used as evidence of expressed genes to improve the gene predictions. High quality gene models ≥300 bp long that had MAKER's AED (annotation edit distance) scores of <0.1 were selected for further analysis. Sequences with AED scores ≥0.1 were also selected if longer than 300 bp and had a BLAST hit with e-value of ≤1e^−20^ to sequences in at least one of the public databases (RefSeq plant RNA, RefSeq plant protein and Swiss-Prot protein). MAKER also provided protein translations of the predicted gene models. These were compared to NCBI's RefSeq plant protein database to detect any frame shift error. The gene models were also compared to the oil palm genome [25] using BLASTN with an e-value cutoff of 1e^−20^. The top hit was used as the putative location of the gene in the genome.

### Gene tagging and coverage

Oil palm BAC was used to estimate the percentage of genes tagged by the methylation filtration (MF) method. This involved, firstly, repeat masking of the EG gene transcripts and then performing iterative subtractive hybridization, in which the transcripts were searched against the BAC genes with a stringent e-value of 1e^−20^. Each iteration used the top hit to mask the BAC gene sequence. The BLAST-and-mask-top-hit iteration was carried out until no further hits were obtained. The BAC gene models predicted by MAKER and sampled by the transcripts were then indicated and quantified by the masked sequences that had been subtracted. To further bracket the estimation of gene space sampling and minimize the number of false positive BAC gene predictions, a smaller BAC gene space represented by alignments of the BAC to known plant RefSeq proteins was determined. The locations where the plant RefSeq proteins aligned were used to calculate the “RefSeq Gene Estimates”.

### Comparison of GT sequences to EST and transcriptome sequences

Sanger EST reads from MPOB and Genbank, and 454 EG transcriptome contigs from two recent oil palm publications [15], [16] were compared to the EG and EO genomic sequence assemblies using BLASTN. The EST and transcriptome sequences were also assembled using CD-HIT-EST [39] at a sequence identity threshold of 0.95 and maximum unmatched percentage of 0.05 to form non-redundant EST clusters. The EST clusters were compared to the GT assemblies using BLASTN. An EST was considered tagged if the match had an e-value of not more than 1e^−20^.

### Global comparison and identification of conserved genes

*Arabidopsis* and date palm genes were downloaded from The Arabidopsis Information Resource (TAIR; http://www.arabidopsis.org/) and WCMC-Q (http://qatar-weill.cornell.edu/research/datepalmGenome/download.html) websites, respectively. The genes were compared to the oil palm unique sequences (contigs and singletons) using TBLASTN. Comparisons of the *Arabidopsis* to date palm genes and *vice versa* were carried out using BLASTP. Reciprocal best BLAST hit method was used to determine potential orthologs. The date palm and oil palm potential orthologs were annotated via BLASTP and BLASTX analysis respectively to Genbank's non-redundant (nr) protein database. Ortholog pairs were annotated as known genes if at least one of the pair had significant similarity to a known gene. All analysis was performed at an e-value cutoff of 1e^−20^.

### Gene ontology (GO)

The GO terms which arose from the BLAST results (searches against UniProt and NCBI databases) and InterProScan [40] were mapped onto the Plant GO Slim annotations using CateGOrizer [41] (formerly known as *GO Terms Classifications Counter*) and the occurrences counted using the single occurrence count option. Predicted genes were functionally annotated via BLAST searches against NCBI RefSeq plant, *A. thaliana* mRNA, *Oryza sativa* mRNA and Swiss-Prot protein databases. All contigs, as well as their corresponding predicted transcripts were also annotated with protein domain and other related information using InterProScan.

### Microsatellite

Microsatellite analysis was carried out using Sputnik (http://espressosoftware.com/sputnik/index.html). A microsatellite had to be at least six di-, five tri- or four tetranucleotide repeat-units long. One imperfection every 10 repeats was allowed.

### Single nucleotide polymorphism (SNP)

Overlapping reads were identified from ACE files by using a Python script. Each nucleotide position in the alignment was interrogated and putative SNP identified only when each allele was supported by at least 2 separate reads. SNP density was calculated by dividing the total number of SNPs by the length of regions with sequence coverage between 4 and 30.

### Transcription factors (TF)

TF of *A. thaliana*, *O. sativa* subspecies indica and japonica, *Triticum aestivum* as well as *Vitis vinifera* in PlantTFDB [42] were downloaded. Oil palm gene models were compared to the PlantTFDB sequences using BLASTP (cutoff: 1e^−20^) and validated using HMMPfam. They were characterized as TF if they had a significant hit to the PlantTFDB sequences and contained at least one of the key domains of their respective TF family.

### Resistance genes

Plant resistance (R) genes were downloaded from PRGdb (http://prgdb.crg.eu/) [43] and converted to a BLAST database. The downloaded R genes were also classified into six classes, as per Yun [44] and Song et al. [45]. The first class, R gene Pto was classified based on the presence of the kinase protein domains [46], [47] while class 2 R genes contain CC (coil-coiled) –NBS (nucleotide binding site) –LRR (leusine rich repeat) protein domains [48]. R genes that contain the TIR (toll interleukin receptor)-NBS-LRR domains was classified as class 3 [49]. In class 4, the genes contain LRR–TM (transmembrane) [50] while class 5 R genes contain the LRR-TM-kinase domains [51]. The last category, uncategorized R genes, are those with domains that cannot be grouped into any of the above mentioned classes [51]. For each class, the R genes were aligned and a HMM model was generated using HMMER [52]. The HMM models were used to identify oil palm R gene homologs, which were validated via BLASTP comparison to RefSeq and the downloaded R genes, and domain search via InterProScan. An e-value cutoff of 1e^−20^ was used for the BLASTP analysis. Information on the protein domains and their locations were used to define the domain signature of each class. For classes 4 and 5, TMHMM (Transmembrane HMM) [53] analysis was carried out to identify transmembrane regions. R genes were clustered using ClusterW [54] prior to phylogenetic analysis using MEGA5 [55].

### microRNAs

The EG and EO contigs were searched against the whole hairpin sequences of miRBase [56] using BLAST. Regions of the contigs with full-length match and few mismatches - typically with 95% identity to microRNAs (miRNAs), were considered as perfect matches. However, regions with very similar but imperfect matches (≥85% similarity; score ≥100), had their secondary structures predicted using the Vienna package [57]. The secondary structure of both the stem-loop and the sequence around the hit region were predicted using RNAfold [58]. The predicted structures were aligned with the RNAdistance program. If the structure around the match showed similarity to the loop, it was considered as a partial match. Mature miRNAs were predicted using MatureBayes [59].

## Results and Discussion

### Assembly of *E. guineensis* and *E. oleifera* sequences

A total 461,286 methylation-filtered and UF sequences were generated from 246,801 plasmids from the respective EG and EO libraries (Table 1). The sequences were analysed and filtered prior to sequence assembly. Methylation-filtered and UF sequence data were combined to improve the quality of both *E. guineensis* (306,558 EGs) and *E. oleifera* (154,728 EOs) assemblies. An additional 559 DNA sequences (434 EGs and 125 EOs) from Genbank were also included in their respective assemblies, mainly to increase the number of SNPs detected.

**Table 1:** Assembly statistics of EG and EO genomic sequences.

Assembly	EG01	EO01
**Description**	EG genomic sequence	EO genomic sequence
**Input:**		
Reads(clones)	306,558(164,224)	154,728(82,577)
Public	434	125
**Result:**		
No. Contigs	45,370	18,836
No. Singletons (≥50 bp)	155,442	92,446
No. Singletons (<50 bp)	17,405	8,556
Total Unique Sequences	200,812	111,282
Total Length of Unique Sequences (nt)	137,247,669	66,077,552
% Unique are Contigs*	23%	17%
% Reads in Contigs	44%	35%
N50 Length	1,166	1,053
Max Length	8,319	7,186
Mean Length	1,063	909

* Percentage of unique sequences that are represented by contigs.

After quality assessment and a size cutoff of 50 bp, 94.6% (289,587) and 94.7% (146,297) of the EG and EO reads respectively were included in the assembly. For EG, the assembly (EG01) produced 45,370 contigs while 155,442 remained as singletons. The N50 of the assembly was 1,166 bp. The EO sequence assembly (EO01) revealed 18,836 contigs and 92,446 singletons with N50 of 1,053 bp. Table 1 summarizes the statistics of EG01 and EO01. These sequences represent an important resource for the research community, especially since there are only limited numbers of oil palm genomic sequences targeting coding regions in the public domain. The collections of sequences are available for download at http://genomsawit.mpob.gov.my and have been registered at NCBI under the *E. guineensis* and *E. oleifera* BioProject accessions PRJNA217845 and PRJNA217846 respectively.

### Size of genome space sampled by methylation filtration

**Figure 1:**
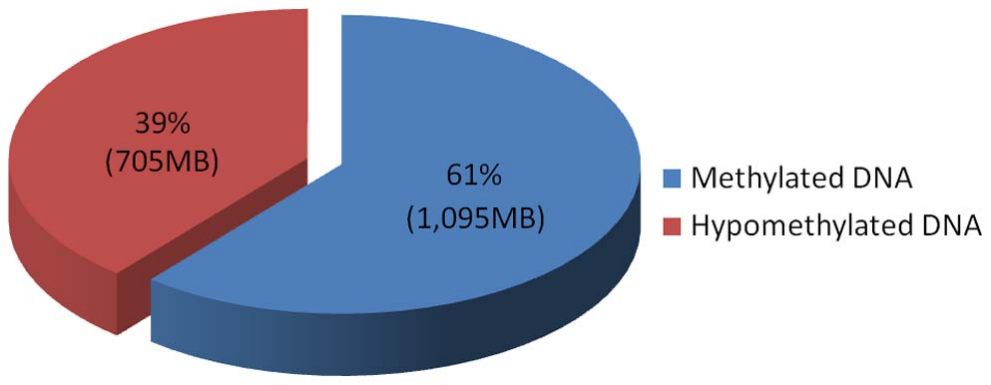
Hypomethylated regions of the oil palm genome sampled by GT Technology. MF reduced the oil palm genome by 61%, thereby allowing sampling of 705 Mb of the hypomethylated region while filtering out 1,095 Mb of the 1,800 Mb genome.

Previous studies in maize [28], [60], [61], land plants [62], sorghum [29], cowpea [63] and *Oryza glaberrima* [64] have shown that MF enriches for sequences from the gene space. Using the method of Bedell et al. [29] (Materials S1), the filter power (FP) of EG was estimated as 2.0 to 2.8, and 2.5 to 2.6 for EO. Based on the estimated FP, the sampled genome size was 643 Mb to 900 Mb for EG, and 692 Mb to 720 Mb for EO. Taking the average FP of EG as 2.4 (the range was 2.0 to 2.8), the estimated hypomethylated space of EG is 705 Mb, similar to the size of the *Sorghum bicolor* genome (Figure 1). Similarly, EO had an average FP of 2.6 and a genome space of 692 Mb. These represent a 2.5 (EG) and 2.6 (EO) fold reduction from the original palm genomes.

The estimation of FP on the EG sequences was supported by a second method, a modification of the technique described by Whitelaw et al. [28] using the formula of Lander and Waterman [34]. Modifications were made for the GT assemblies used, where unlike previously, the MF and UF reads were assembled together to obtain contigs. The Lander Waterman formula was adapted to obtain the number of islands expected in a mixed assembly of sequences obtained by sampling from two genomes of different effective sizes. This was used to infer the size of the filtered genome, given the known size of the unfiltered genome and the number of islands observed in the mixed assembly. Using this approach, the genome space estimated for EG was 563 Mb with a FP of 3.2. In EO, the sequence coverage was insufficient to perform the analysis. The genome sampling method requires at least 0.1x coverage of the genome to be reliable. Nevertheless, both methods showed that the MF libraries had FP of at least 2.0. Gene enrichment in the MF sequences of oil palm was similar to the 2.47 to 2.83-fold enrichment reported in soybean, potato and oilseed rape, and higher than the 1.89-fold enrichment in rice [62].

### Gene models

The oil palm gene models were predicted using MAKER, an evidence-based gene prediction tool. MAKER uses a combination of gene prediction software along with alignments to known transcripts and proteins in producing high quality gene predictions. A total 80,297 gene models were predicted for the EG01, EO01 and BAC contigs. Considering only the high quality transcripts, the number was reduced to 5,504 (166 in BAC, 3,954 in EG01 and 1,385 in EO01 contigs) (File S1). The predicted transcripts were searched against the recently released oil palm genome build [25] to determine whether GT genes were represented in the chromosomes. A total 3,934 EG01 and 1,375 EO01 gene models were successfully placed (Table 2). Of these, 3,048 EG01 and 1,049 EO01 gene models were identified on the 16 oil palm chromosomes [25]. The predicted genes were evenly distributed on all the chromosomes. The location of the GT sequences could help to pinpoint the exonic regions of the oil palm genome.

**Table 2:** Identification of GT gene models in the oil palm EG5 chromosomes.

EG5 Chromosomes	Predicted Transcripts	
	EG01	EO01
EG5_Chr1	369	139
EG5_Chr2	297	106
EG5_Chr3	296	106
EG5_Chr4	238	91
EG5_Chr5	248	75
EG5_Chr6	171	50
EG5_Chr7	178	79
EG5_Chr8	175	60
EG5_Chr9	138	51
EG5_Chr10	174	57
EG5_Chr11	131	33
EG5_Chr12	151	45
EG5_Chr13	121	36
EG5_Chr14	145	44
EG5_Chr15	126	42
EG5_Chr16	90	35
Other scaffolds	886	326
Total hits	3934	1375

### Gene tagging and coverage

Oil palm BAC were used to estimate the percentage of genes tagged by MF. Full length repeat-masked EG01 contigs and singletons were subtracted *in silico* from the BAC gene models using the iterative BLAST method. The BAC gene sequences sampled were then indicated and quantified. The analysis showed at least 62% of the gene space on the BAC was sampled. The analysis is indicated in Table 3 as the “Predicted Gene Estimates”.

**Table 3:** Estimates of percentage BAC gene space sampled.

Estimated % Gene Space Sampled	Pool A*	Pool B*	Pool C*	Pool D*
Predicted Gene Estimates	77%	79%	62%	71%
RefSeq Gene Estimates	71%	77%	68%	66%
Masked Sanger EST contigs and singletons	33%	36%	34%	31%
(25,781 sequences, 15 Mb)				
Masked 454 transcriptome	36%	35%	40%	47%
(70,729 sequences, 69 Mb)				

* Pool A, B, C and D (∼44 BAC/pool) are equimolar pools representing ∼10 megabases of the oil palm genome.

Steps were taken to minimize false BAC gene predictions as these can inflate the gene space. For EG01, this was done by sampling smaller BAC gene spaces, represented by alignments of BAC to known plant reference sequence proteins. BAC were positionally annotated with known plant RefSeq proteins and 157 BAC sequence transcripts corresponding to these gene positions extracted (Table 4). The same stringent *in-silico* subtractive hybridization of full length repeat-masked EG01 contigs and singletons subtracted from this reduced set of BAC gene transcripts resulted in the “RefSeq Gene Estimates” in Table 3.

**Table 4:** Reduced BAC gene space annotated by plant RefSeq orthologs.

	Pool A	Pool B	Pool C	Pool D
No. transcripts	49	46	27	35
Mean transcript length	1,145	1,059	1,150	992
Maximum transcript length	3,423	2,934	3,642	2,100

To evaluate gene space coverage, EG01 sampling of this reduced space was compared with that achieved by two other gene sampling methods, EST and 454 transcriptome sequencing. EST contigs assembled from Sanger EST reads (MPOB and Genbank), and 454 EG transcriptome contigs [15], [16] were repeat masked and compared to the reduced set of BAC gene transcripts using the same iterative method described above. The results showed that the gene space sampled in EG01 was higher than that obtained using EST sequencing and from the 454 sequenced transcriptome. This shows the efficiency of the method for gene discovery. Nevertheless, it is important to note that the number of ESTs and transcriptome data were low and specific only to the tissues sampled. Most of the ESTs [6], [7], [9], [65] were sampled from tissue culture materials while the transcriptome data was mainly sampled from mesocarp tissues [15], [16]. The BACs were randomly sampled and genes expressed in these tissues might not be represented in these BACs. This could have resulted in the low level of BAC gene space sampled by the transcripts.

### Comparison of GT sequences to EST and transcriptome sequences

**Figure 2:**
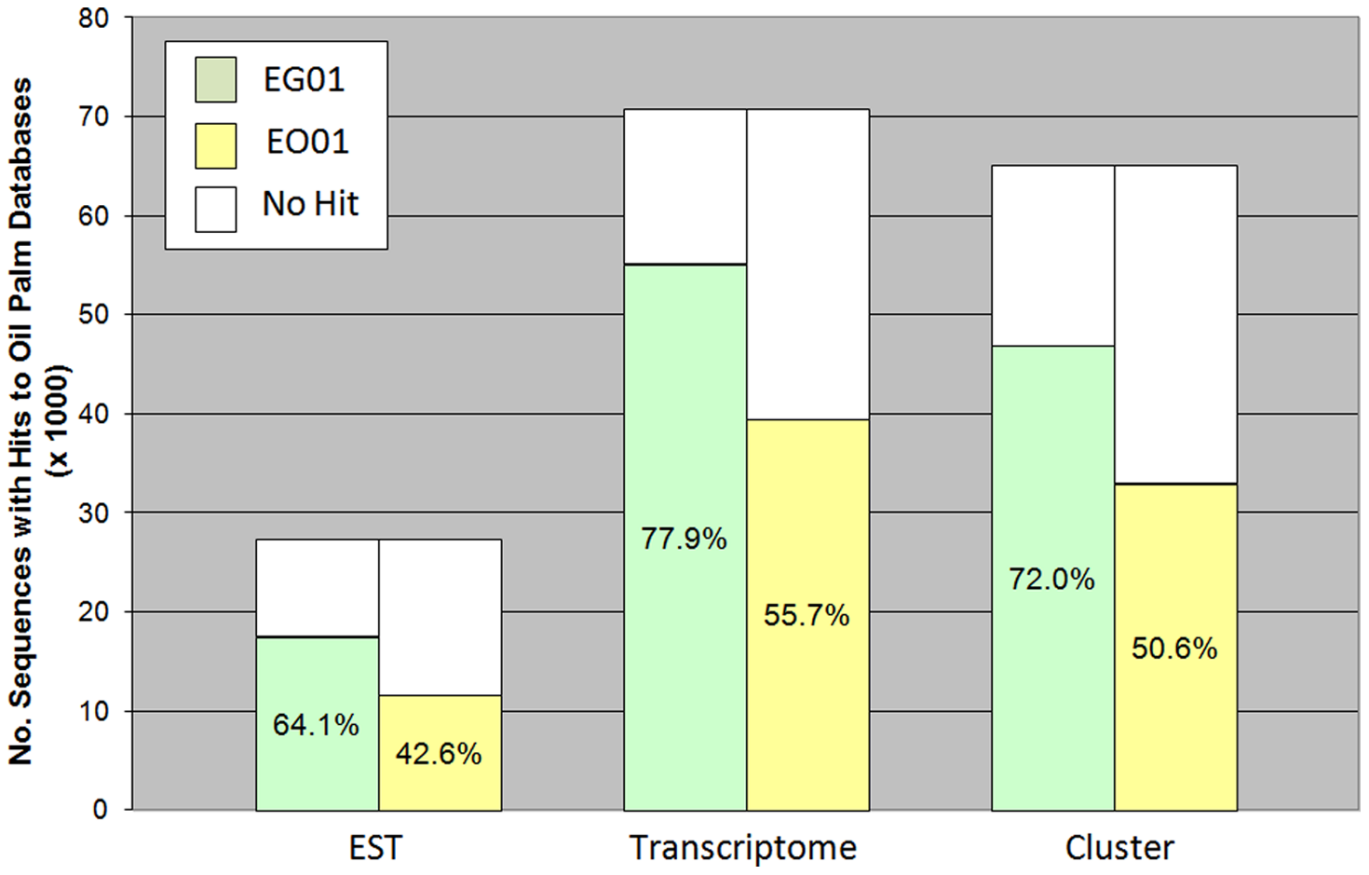
BLASTN analysis of oil palm EST and transcriptome sequences to EG01 and EO01. The percentage of EST, transcriptome and Cluster sequences that have significant similarity (≤1e^−20^) to EG01 and EO01 sequences are shown in green and yellow respectively. Cluster is a set of non-redundant sequences generated from the assembly of the EST and transcriptome data by CD-HIT-EST.

In order to determine whether the available oil palm ESTs were tagged by the MF sequences, the GT assemblies were compared to the EST and transcriptome contig sequences. EG01 was able to tag between 64% and 78% of the EST sequences. Nevertheless, to obtain a better estimate of the number of ESTs tagged, the GT assemblies were compared to a non-redundant set of ESTs. The analysis showed that EG01 sequences were able to tag a high percentage of the EST clusters (72%). The results obtained were comparable to those from cowpea, where 73.7% of the EST dataset matched the GT sequences [63]. In EO01, the percentage was lower since the EST and transcriptome sequences were mostly obtained from EG. Differences between the two oil palm species most likely accounted for the reduced similarity. Figure 2 show the percentages of EST and transcriptome sequences that have hits to EG01 and EO01 sequences. Interestingly, comparison of the EG01 and EO01 gene models showed that 23.3% and 21.4% of the gene models respectively, were absent from the EST and transcriptome data (Table 5). The GT sequences not only had a high coverage of the available EST sequences, it was also able to identify additional genes that would be an important resource for research. The list of genes not tagged by the EST data is in File S2.

**Table 5:** Comparison of predicted oil palm gene models against EST and transcriptome data.

Data Set	Predicted Gene Models	Significant Hit*	No Hit*
EG01	3954	3034	920
EO01	1385	1088	297

* e-value cutoff: 1e^−20^.

### Global comparison of EG01 sequences to Arabidopsis and date palm genes

The oil palm genomic sequences were compared to *Arabidopsis* and date palm genes to determine the coverage of the genes tagged by the EG01 data. The *Arabidopsis* and date palm dataset contains 35,386 [66] and 28,890 [67] genes respectively. In the first analysis, the *Arabidopsis* sequences were used as TBLASTN queries against EG and as BLASTP queries for the date palm protein sequences. There were 15,431 (44%) and 24,604 (70%) *Arabidopsis* genes with matches to oil palm and date palm genes, respectively. The fewer hits to oil palm were not surprising considering the low coverage of its GT sequences in this study. As date palm is the closest related plant genome to oil palm to be sequenced, the analysis was repeated using date palm genes as query in searches against oil palm EG and *Arabidopsis* protein sequences. This provided a better representation for cross species gene annotation.

In the analysis, 17,838 (62%) date palm sequences had hits to oil palm while 19,489 (68%) had matches to *Arabidopsis*. A total 371 date palm proteins had matches to EG but not to *Arabidopsis*. Reciprocal best BLAST hits of these sequences identified 192 potential orthologs of date palm and oil palm that did not have any similarity to *Arabidopsis* genes. Comparison to Genbank's nr protein database showed that five of these genes had similarity to repeat elements and were removed from further analysis. About 50% of the putative orthologs did not have any similarity to sequences in Genbank. The remaining sequences had hits to known [58] and uncharacterized [34] genes, such as hypothetical genes or putative proteins, of which 19 had significant similarity only to monocotyledon genes in the nr database. The list of these orthologs is available in File S3. These genes may be useful to study conserved functions between oil palm and date palm. Overall, even at low coverage, the GT method was able to tag a high percentage of genes in the date palm genome and identify genes that are conserved between date palm and oil palm.

### Gene ontology (GO)

BLAST results from the functional annotations analysis (specifically, searches against the UniProtKB and RefSeq plant mRNA databases) of the good quality gene models were searched against the UniProt and NCBI databases for GO terms using a set of custom scripts. Final results of the non-redundant GO analysis were merged based on results from the BLAST and InterProScan searches. Table 6 shows the domain annotation and summary of the GO search results. The GO analysis results indicated that the predicted genes were distributed in different functional classes (Figure S1). More importantly, similar trends were observed in both EG and EO functional classification, although, as expected there were differences in the number of genes in each functional class. The three top level categories were Molecular Function (ML), Biological Process (BP) and Cellular Component (CC). It is worthwhile to note that 75% of the predicted genes in EG were assigned to ML, while 43% and 41% were categorized with BP and CC terms, respectively. Notably, a similar trend was observed in EO. The results also showed that 18% of the predicted genes could not be annotated with GO terms. Analysis of their annotation showed that more than 50% of them had similarity to hypothetical proteins.

**Table 6:** Summary of domain, sub-cellular localisation and GO annotation.

Dataset	EG01 Contigs	EO01 Contigs	BAC Contigs
Predicted Genes with Domain annotations	2,861	1,013	86
Predicted Genes with SignalP predictions	581	183	n/a
Predicted Genes with TargetP predictions	148	48	n/a
Predicted Genes with GO Molecular Function terms	2,960	1,068	129
Predicted Genes with GO Biological Process terms	1704	636	96
Predicted Genes with GO Cellular Component terms	1623	622	59

A more comprehensive insight into ML revealed that the top subcategories for EG and EO were molecular function [GO:0003674], catalytic activity [GO:0003824] and transferase activity [GO:0016740]. The majority of the predicted genes annotated under the GO term BP category were regulating transcription, DNA-dependence [GO:0006355] and proteolysis [GO:0006508]. Interestingly, the GT data did not have over-representation of highly expressed genes, such as ribosomal genes, as seen in the ribosomal and cytoskeletal peaks in the BAC data in Figure S1c. This suggests that the GT sequences were randomly distributed in the hypomethylated regions of the genome. The gene ontology results are given in File S1.

The gene models (3,954 EG and 1,385 EO) were also mapped onto the Kyoto Encyclopedia of Genes and Genomes (KEGG) orthology database [68], which enable reconstruction of the KEGG pathways. A total 488 EG01 genes were assigned to KO (KEGG Ortholog), of which 317 were mapped onto 71 pathways. As for EO, out of 223 KO annotated genes, 161 were successfully assigned and mapped onto 56 pathways. In EG, oxidative phosphorylation [25], ribosome [23] and glycolysis/gluconeogenesis [16] are the most abundant. A similar result was observed in EO, where oxidative phosphorylation [28] represented the most common pathway, followed by photosynthesis [15]. Table S2 shows the number of genes categorized into KEGG pathways.

### Microsatellites

**Figure 3:**
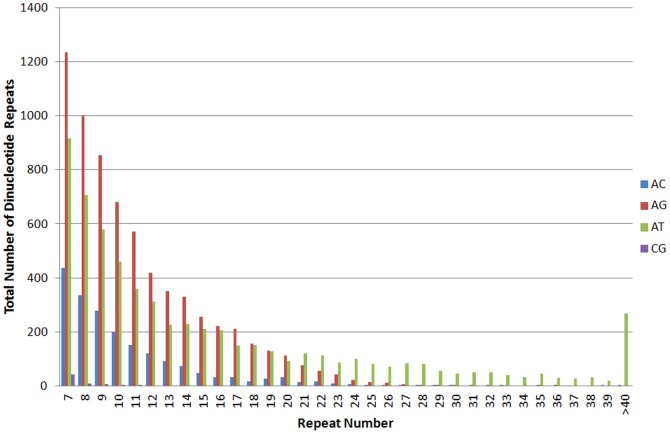
Distribution of dinucleotide repeats observed in EG01 SSR. The AC, AG, AT and CG repeats are represented in blue, red, green and purple respectively. The total number of observations for each repeat are represented by the height of the respective column.

Microsatellites, also known as Simple Sequence Repeats (SSR), are important sources of molecular markers for genetic studies. The earliest exploitation of a sizeable number of EG genomic SSR for genetic mapping was by Billotte and colleagues [69] with 369 SSR of dinucleotide (GA and GT) and trinucleotide (CCG) repeats used. Although the researchers were able to show the effectiveness of SSR as molecular markers, the number of SSR used was small. Hence the GeneThresher sequences were mined for di-, tri- and tetranucleotide repeats, where 23,621 and 10,131 SSR were identified from EG01 and EO01, respectively (Table 7). The dinucleotide repeats in the assembled sequences of both oil palm species exceeded those of tri- and tetranucleotide repeats. This was consistent with what has been reported earlier by Tranbarger et al. [70] who found that dinucleotide repeats were the most abundant EST-SSR (36%) in oil palm followed by tri- (24%) and tetra- (29%) motifs. The most frequent dinucleotides in their transcriptome data were those with 6 repeat motifs, compared to 17–18 repeat motifs in the genomic SSR reported by Billotte et al. [69], [71]. The authors concluded that there was a higher frequency of lower number of repeat motifs in the coding region. The current MF data reflected this pattern as it also mainly covered the genic regions. Nevertheless, the MF data also revealed a high percentage of AT dinucleotide with 40 repeat motifs (Figure 3). This suggests that the MF sequences also contained sequences from non-genic regions, most likely the flanking regions of genes.

**Table 7:** Summary of di-, tri- and tetranucleotide repeat motifs in EG01, EO01 and BAC.

Data Set	Dinucleotides	Trinucleotides	Tetranucleotides	Total
EG01	14, 910	5,152	3,559	23,621
EO01	6,366	2,247	1,518	10,131
BAC	594	328	247	1,169

Among dimerics in EG01, the AG motif was the most abundant repeat with 28.62%, followed by AT (26.06%) and AC (8.18%) respectively (Table S3). A similar trend was also observed in EO01 (Table S4). Low et al. [7] identified similar trends in dinucleotide repeat motifs and repeat numbers in oil palm ESTs with AG/CT (67%) and AT (21%) the most abundant, followed by AC/GT (11%) and CG (0.3%). The AG/CT dinucleotide repeat motif was consistent with the high frequencies in the genic regions of *A. thaliana* [72] and rice [73]. Similar patterns were also noted for EST-SSR in peanut [74] and cacao [75]. The CG motif was generally in low abundance in both *Elaeis* species.

Although the trinucleotide repeats are not the most prevalent SSR in the hypomethylated regions of oil palm, they are of interest as they are found predominantly in the exonic regions. Low et al. [7] compared the distribution of a small number of oil palm full-length EST-SSR and found the mono- and di-nucleotide repeats in the untranslated regions (UTR), whereas trinucleotides were in both UTR and open reading frames (ORF), with a preference for ORF. Zhang and colleagues [72] observed that the trinucleotides, followed by the hexanucleotides accounted for 92.6% of the SSR in the coding regions of *A. thaliana*. A similar observation was also made by Toth et al. [76] that trimers and hexamers were rampant in the exon regions of eukaryotic genomes. In EG01 and EO01, the most abundant trimers were AAG (5.94%, 6.11%), AAT (5.86%, 5.89%), AGG (3.29%, 3.35%) and CCG (1.99%, 2.06%), respectively (Tables S3 and S4). The trend is similar to the patterns observed by Low et al. [7] in oil palm ESTs, where the most prevalent trinucleotides were AAG/CTT (23%), AGG/CCT (13%), CCG/CGG (11%) and AAT/ATT (11%). Although the most prevalent trimer in both the genomic and EST-SSR was AAG, the genomic sequences had a higher representation of AAT repeats compared to EST-SSR. The high abundance of the tri-repeat motif of AAG in EG and EO was similar to that reported in the EST sequences of cotton (*G. hirsutum*, *G. arboretum* and *G.* raimondii) [77] and cucumber [78], respectively. A slightly different frequency of repeat motifs of AAG and AAC was observed in the exonic regions of embryophyta [76]. Zhang et al. [72] also observed that the AAG motif was the most prominent repeat in the 5′UTR region of *A. thaliana*.

In the hypomethylated region of *Elaeis*, the trimer motif CCG was also observed albeit at low percentage of 1.99–2.06%. This is different from other monocots (maize and wheat), where the CCG motif alone accounted for half of the trinucleotide repeats in rice and is also moderately rich in other plants [79]. Yonemaru et al. [80] also found that the most frequent trimers in *S. bicolor* were CGC/GCG. This shows that diverse taxonomic groups exhibit different tendencies for SSR types which are also influenced by their genomic locations [76]. In the 33 different tetranucleotide repeat motifs found in the *Elaeis* sequences, AAAT motif was the most frequent, followed by AAAG and ACAT. Similar motifs were also found in the *A. thaliana* genome, which is AT-rich [81], and in the genome of cotton *G. raimondii* [77].

The MF sequences proved to be an important source of SSR markers. In fact, the utility of SSR from methylation filtered sequences for oil palm genetic diversity analysis and genetic mapping was demonstrated by Zaki et al. [30] and Ting et al. [31], respectively. The SSR markers from MF sequences have several advantages. One is that they show increased polymorphism associated with genomic based SSR markers compared to EST-SSR. At the same time, since they represent mostly genic regions, they can be used to target candidate genes, similar to EST-SSR. The locations of SSR in EG01 and EO01 are given in File S4.

### Single nucleotide polymorphism

Evolution of the techniques for identification of molecular markers in recent years has led to the discovery of SNPs, which is a single base difference in a DNA sequence with an alternative of two possible nucleotides at a specific location on the chromosome [82]. Initially, the number of putative SNPs identified was 36,138 for EG01 and 14,640 for EO01 contigs. However, to avoid false positives, SNPs with extraordinarily high coverage depth (>30; 2 standard deviation from mean) were excluded from further analysis. This removed SNPs in repetitive or duplicated regions of the genome, leaving 28,842 and 12,578 for EG and EO, respectively. The list of SNPs in EG01 and EO01 are available in File S4. The SNP densities were 2.30 and 2.83 per 100 bp for EG and EO, respectively. However, a previous study on oil palm ESTs showed a density of 1.36 SNPs per 100 bp [83]. As the SNP density in the genic region is expected to be lower than the non-genic region, it is likely that the GT SNP densities are not reflective of the SNP densities of the oil palm genome. The sequence coverage needs to be increased to have a better estimate of the SNP density. Sequence depth values across all contigs for EG and EO are provided in Figure S2. The depths varied widely as most positions within most contigs are only supported by a single read.

The SNPs were grouped into either transition (C/T or G/A) or transversion (C/G, A/T, C/A or T/G) nucleotide substitutions. The frequency of transition exceeded transversion (Table 8), similar to that reported for 1,317 SNPs mined from 5,452 oil palm sequences from seven tissues [83]. Similar trends were observed in maize [84], *S. bicolor* [85] and ginger [86]. In Table 8, the number of SNPs observed in the EG and EO contigs for both transition type SNPs (G/A and C/T) showed no significant difference. However, in transversions, the A/T type SNPs were more frequent than other transversions, and collectively accounted for 44.2% (EG) and 41.3% (EO) of all transversions. The overall transition *vs* transversion ratio in EO was 7.52, which indicates higher transitions over transversions. In EG, the ratio was slightly lower (7.17), consistent with Riju and Arunachalam [87] who identified an overall EO transition *vs* transversion ratio of 1.40 for EO and 1.02 for EG. They opined that the transition *vs* transversion rate is important to understand DNA evolution, with a low value indicative of high genetic divergence and *vice versa*. Interestingly, the lower divergence of EO *vis-à-vis* EG had been demonstrated experimentally using various marker systems, such as SSR [31], [88] and even a small number of SNPs [89].

**Table 8:** Summary of SNPs.

	EG01 Contigs	EO01 Contigs
**Transitions**		
C/T	12,391	5,638
G/A	12,397	5,464
**Transversions**		
A/T	1,928	866
C/G	180	97
G/T	696	226
A/C	650	287
Total	28,242	12,578

In barley [90], higher polymorphism rate was observed for transition SNPs (71%) *vs* transversions (29%), information of possible importance for identification of informative markers. Categorizing the SNPs into transition and transversions could potentially improve efficiency and reduce the number of non-polymorphic SNP markers. Furthermore, as the GT data represent exonic regions of the genome that encode for genes and their regulatory regions, identification of non-synonymous SNPs in genes associated with traits could potentially provide insight into the modulation of the trait. This was recently reported when an important monogenic trait (SHELL) in oil palm was shown to be caused by two independent SNPs in a single gene. The mutations disrupt the DNA-binding domain of a MADS-box gene homologue of *SEEDSTICK*, resulting in three different EG fruit forms - *dura*, *pisifera* and the hybrid *tenera*. This single gene is responsible for the hybrid vigour or heterosis observed in the *tenera* fruit form of oil palm [91]. The polymorphisms in the gene will prove to be an important diagnostic assay for commercial seed production and to enhance breeding activities in oil palm.

### Transcription factors

Transcription factors (TF) help regulate gene expression and are an integral part in the development of an organism. The number of TF in plant genomes is large - 6 to 9% of the coding regions that code for TF [24], [42], [92], [93]. Analysis of *Musa acuminata* genome recently showed that 8.6% or 3,155 of its protein-coding gene models coded for TF. This represents one of the highest numbers of TF identified in a sequenced plant genome [93]. The evolution of a large number of TF could explain the diversity and complexity observed in plants. In oil palm, comparison of the EG01 and EO01 gene models to TF from *A. thaliana*, *O. sativa*, *T. aestivum* and *V. vinifera* from the PlantTFDB database [42] showed that both libraries contained 37 TF gene families, while the BAC sequences were able to identify two additional gene families. A total of 178 and 61 transcriptional factors were identified from the EG01 and EO01 gene models, respectively. The numbers of GT sequences for each gene family are listed in Table 9.

**Table 9:** Oil palm TF in EG01, EO01 and BAC sequences.

Transcription Factor	EG	EO	BAC	Transcription Factor	EG	EO	BAC
AP2	4	1		GRF	2		
ARF	7	2		HB-other	1		
ARR-B	2	1		HD-ZIP	12	5	
BBR/BPC	2			HSF	1		
BES1	1			LBD	4		
bHLH	12	6		M-type	1		
bZIP	8	6		MYB	12	5	
C2H2	16	3		MYB_related	2		
C3H	5	2		NAC	10	4	
CAMTA	1			NF-X1	1		
CO-like		1		NF-YB	3	1	
CPP	1	1		Nin-like			1
Dof	7			RAV	1		
E2F/DP		2		SBP	3	1	
EIL		1		SRS	1		
ERF	13	8	1	TALE	10	1	
FAR1			1	TCP	4	1	
G2-like	6	2		WOX	1		
GATA	2	1		WRKY	7	2	
GRAS	15	4		**Total**	**178**	**61**	**3**

Ethylene Response Factor (ERF) is the one of the biggest group of TF identified. This is not surprising as the ERF family is the most abundant TF in PlantTFDB and second most abundant in DRTF (rice transcriptional factor database) [94]. The analysis also revealed five Apetala2 (AP2) and one RAV genes. These three gene families belong to the AP2/ERF TF superfamily involved in responding to plant biotic and abiotic stress [95], [96]. The ERF sub-family is also known for its involvement in regulating the expression of pathogenesis-related (PR) genes and could play a role in the transduction of various signals to a suite of downstream defence genes [97]. These genes are important for studies related to how oil palm defends itself against pathogens, especially the fungus *Ganoderma*, which is the cause of a major oil palm disease in Malaysia and Indonesia.

Analysis of the GT sequences also revealed TF associated with floral development and tissue culture, such as homeodomain proteins, MADS, Squamosa (SBP) and Apetala2 (AP2). These genes are involved in floral organ patterning and are expressed in different stages of floral development [98]. In Arabidopsis, AP2 is required for the specification of the first and second whorl organ identities [99]. MADS box genes are also hypothesized to be involved in clonal abnormality, namely mantled flowers in ramets [100]. Although clonal abnormality in oil palm has been associated with changes in methylation, the role of MADS box genes in this phenomenon is being investigated. In fact, methylation changes in this group of genes that determine the ABC model for floral development could be pivotal in the clonal abnormality phenomenon observed [101]. In this study, a MADS box gene was identified in the GT dataset. An additional putative MADS box gene with significant similarity (5e^−27^) to a MIKC gene but only containing the k domain was also identified. The sequence did not contain the MADS domain and was thus not included in Table 9.

The TF dataset represents an important resource not only to study floral development and stress responses but also other important mechanisms in oil palm. Another important application of the TF data is that 58 of these sequences contained SSR or SNPs, which can help localize the genes on the oil palm genetic maps. As some genes of similar function tend to cluster in the genome, identification of the genetic loci would allow researchers to test other genes/markers flanking the TF for association with specific traits. The list of TF is available in File S1.

### Resistance gene homolog

Fungi cause three major diseases in oil palm - *Fusarium* wilt, bud rot and basal stem rot. Identification of the genes involved in pathogenicity and resistance is an important step towards identifying disease tolerant/resistant palms. As such, efforts have been made to identify oil palm pathogenesis-related genes (R genes homolog) in the EG01 and EO01 gene models. R genes play an important role in the early stages of plant defense mechanism [102], [103]. They have distinct interactions with specific molecules secreted by the pathogen into plant cells during invasion [104]–[106]. Comparison of the predicted amino acid sequences from EG01 and EO01 to known R genes revealed 52 EG and 13 EO R gene homologs (File S1).

Of these, only two class 1 R genes from EG and one from EO containing both the kinase and Pto domains were identified. The analysis also revealed that class 2 was the largest group of R genes identified. Homology search and InterProScan confirmed the presence of 29 EG class 2 R genes. They represent ∼44.6% of the oil palm R genes identified, in line with previous reports that the NBS-LRR group is the largest class of R genes [107]. Barbosa-da-silva and colleagues [50] also reported that R genes with NBS-LRR domain properties are the largest group and contained the most functionally defined R genes. We suspect that this class is an important component of the plant immune response system. The NBS domain is involved in ATP binding and hydrolysis, while the LRR domain is the determinant of response specificity [108]. However, no class 2 R gene was identified in the EO01 data, probably due to the lower coverage of the EO libraries. The R genes in EO01 were probably partial length and categorized as class 6 (uncategorized).

The analysis also did not reveal any class 3 R genes in the EG01 and EO01 data. This was not surprising as class 3 R genes are predominantly found in dicotyledons. The only monocotyledon TIR-NBS-LRR R gene identified was reported in the Triticum-Thinopyrus line [109]. Oil palm, being a monocotyledon, is not expected to have homologues of class 3 R genes. Homologues to class 4, 5 and 6 were also identified. Eight EG01 gene models were classified as class 4 R genes. An additional seven EG01 and three EO01 gene models that contained the LRR-TM-kinase domains were classified as class 5 R gene. The final class of R genes, the ‘uncategorised’, contained 15 gene models.

**Figure 4:**
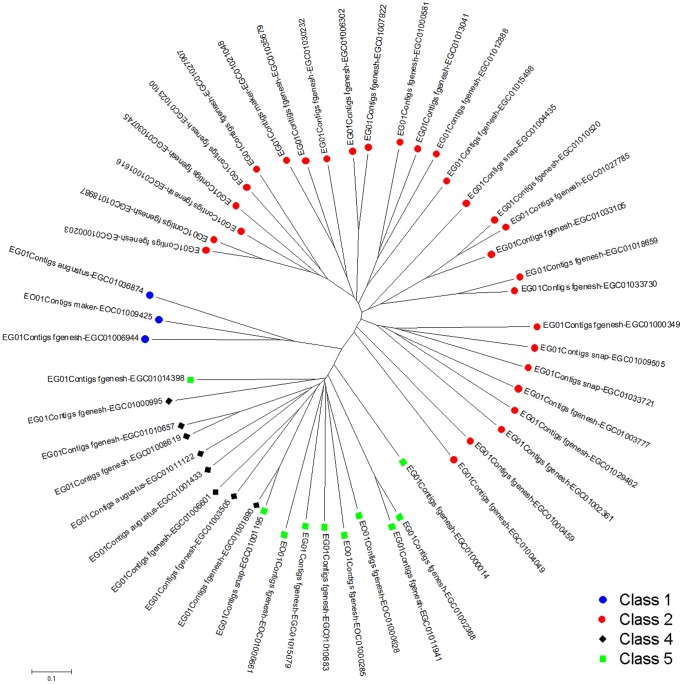
Phylogenetic analysis of EG and EO R genes. Class 1, 2, 4 and 5 are represented by blue, red, black and green circles respectively.

Classification of the oil palm R genes homologs were further verified using phylogenetic analysis (Figure 4). The analysis showed three distinct clades, representing class 1, 2, and a combination of class 4 and 5. Class 4 and 5 share the same clade because both classes contained the LRR and TM domains. Class 5 can be differentiated by an additional kinase domain. Class 6 R genes (uncategorised) were not included in the phylogenetic analysis. The phylogenetic analysis generally concurred with the classification of the genes. The R genes identified in this study would facilitate the understanding of how oil palm defends itself against diseases such as bud rot and basal stem rot, which have devastated large tracts of oil palm plantations. Combining knowledge of R genes and associating it with quantitative trait loci analysis of germplasm/breeding populations for disease resistance [110] would help with future development of elite oil palm varieties.

### microRNAs

miRNAs are short sequences from a class of RNAs ∼18 to 24 nt in length. They are produced by dicer-catalyzed excision from stem-loop precursors and play an important role in diverse organisms [111]. The functional role of miRNAs can be elucidated by the identification of their mRNA targets [112]. At present, most of the plant miRNAs identified belong to the model plant *A. thaliana*. Nevertheless, there is a growing resource of miRNAs from other plants, such as rice, tomato and sorghum [112]–[114]. In this study, a homology approach was used to identify oil palm miRNAs. The EG01 and EO01 contigs were searched against the stem-loop precursors of miRBase [56]. As mature miRNAs are short, using the stem-loop precursors provide a longer sequence for comparison to identify conserved regions.

Forty miRNAs were identified from the contigs. Of them, 28 were predicted in EG01 contigs where 10 contigs gave perfect hits and 18 partial matches. In EO01 contigs, nine gave perfect hits and three partial matches to known miRNAs in the registry. Stringent parameters with 85% similarity cutoff and a score of ≥100 were used to avoid false positives. The list of predicted miRNAs for EG01 and EO01 is shown in Table S5. However, the predictions are dependent on the miRNAs deposited in miRBase. The small number of predicted miRNAs obtained was most likely due to the lack of closely related species in miRBase. The quality of the predicted putative miRNAs was further verified by looking at mismatches in the hit regions. This was to ensure that the mismatches did not break the secondary structure and only fell on the open-loop regions. As a result, 14 predicted mature sequences were retrieved from MatureBayes program as potential oil palm miRNAs (Table 10).

**Table 10:** List of predicted mature miRNAs from EG01 and EO01 contigs.

Contigs	Best Hits with miRNAs in miRBase	Match Status	Predicted Mature miRNA*
EGC01043189	peu-MIR2911	Perfect	gcggcgacccgcucucgccgcg
EGC01002494	peu-MIR2911	Perfect	gcggcgacccgcucucgccgcg
EGC01007640	peu-MIR2911	Perfect	gcggcgacccgcucucgccgcg
EGC01002621	peu-MIR2916	Perfect	ccugaaagcaacauccgccgau
EGC01009851	peu-MIR2916	Perfect	gaagacgaucagauaccguccu
EGC01006056	peu-MIR2911	Perfect	gcggcgacccgcucucgccgcg
EGC01005984	peu-MIR2911	Perfect	gcggcgacccgcucucgccgcg
EGC01029522	ptc-MIR156j	Perfect	ugaugcagagcuccaugcaucc
EOC01000015	peu-MIR2916	Perfect	ugggggcucgaagacgaucagau
	peu-MIR2914		
	peu-MIR2910		
EOC01008865	vvi-MIR319f	Perfect	gaugcaaugggucuugcauguc
EOC01001645	sbi-MIR167g	Perfect	ggcaucgggggcgcaacgcccu
EOC01006693	ptc-MIR319e	Perfect	gcuuccuucagcccacucaugg
EOC01010601	vvi-MIR845a	Perfect	cucauccaagaucuagaggaaa
EOC01007557	vvi-MIR845b	Perfect	cccuucaguccaaucggcgggc

* Mature miRNAs were predicted using MatureBayes program.

Target prediction of the 14 potential miRNAs identified one target mRNA transcript that is similar to the Rab21-family small GTPase, which is a small GTP-binding protein of the Ras superfamily [115]. Identification of only a single target gene is not surprising as most of the oil palm mRNA transcripts available are not full-length and probably lack the UTR regions. Nevertheless, identification of the Rab protein is interesting as it plays an important role in regulating intracellular vesicle trafficking. In plants, Rab proteins have been implicated in transport between the endoplasmic reticulum and Golgi apparatus, trafficking of soluble cargo, fusion of endocytic vesicles and vesicular transport along microtubules. Studies in *Arabidopsis* have also identified that certain Rab proteins are influenced by hormones, such as ethylene and auxin [116]. It would be interesting to determine the expression of the oil palm miRNAs, and its interaction with the Rab21 transcript. Looking forward, the recently released oil palm genome data will provide valuable information for further characterization of the oil palm miRNAs.

## Supporting Information

Figure S1 Gene ontology classification of EG01, EO01 and BAC sequences. Three GO categories, [A] Molecular function (ML) [B] Biological process (BP), and [C] Cellular component (CC) terms were mapped to Plant Slim GO annotations using CateGOrizer.
(DOCX)

Figure S2 **Depth at SNP positions for [A] EG01 and [B] EO01 contigs.** The red line indicates the cut-off of two standard deviation from mean, where the SNPs on the left of the line were defined as unreliable.
(DOCX)

Table S1 ***E. guineensis*** **and** ***E. oleifera*** **filtered and unfiltered genomic library information.**
(DOCX)

Table S2 **Categorization of EG and EO genes into KEGG pathways.**
(DOCX)

Table S3 **Di-, tri- and tetranucleotide repeats identified in EG01.**
(DOCX)

Table S4 **Di-, tri- and tetranucleotide repeats identified in EO01.**
(DOCX)

Table S5 **List of perfect and partial match miRNAs from EG01 and EO01 contigs.**
(DOCX)

File S1 **Gene information and annotations.**
(XLSX)

File S2 **List of genes not tagged by oil palm ESTs.**
(XLSX)

File S3 **List of orthologs.**
(XLSX)

File S4 **List of SSRs and SNPs.**
(XLS)

Materials S1 **Formula to calculate gene enrichment.**
(DOCX)

Materials S2 **Formula to calculate genome sampling.**
(DOCX)
