# Supplementary material for: Analyses of Hypomethylated Oil Palm Gene Space
Source: PLoS One. 2014 Jan 30;9(1):e86728. doi: 10.1371/journal.pone.0086728 (PMC3907425; doi:10.1371/journal.pone.0086728)
Supplement: Figure S1 — Gene ontology classification of EG01, EO01 and BAC sequences. Three GO categories, [A] Molecular function (ML) [B] Biological process (BP), and [C] Cellular component (CC) terms were mapped to Plant Slim GO annotations using CateGOrizer. (DOCX) [file pone.0086728.s001.docx]

**A**

**
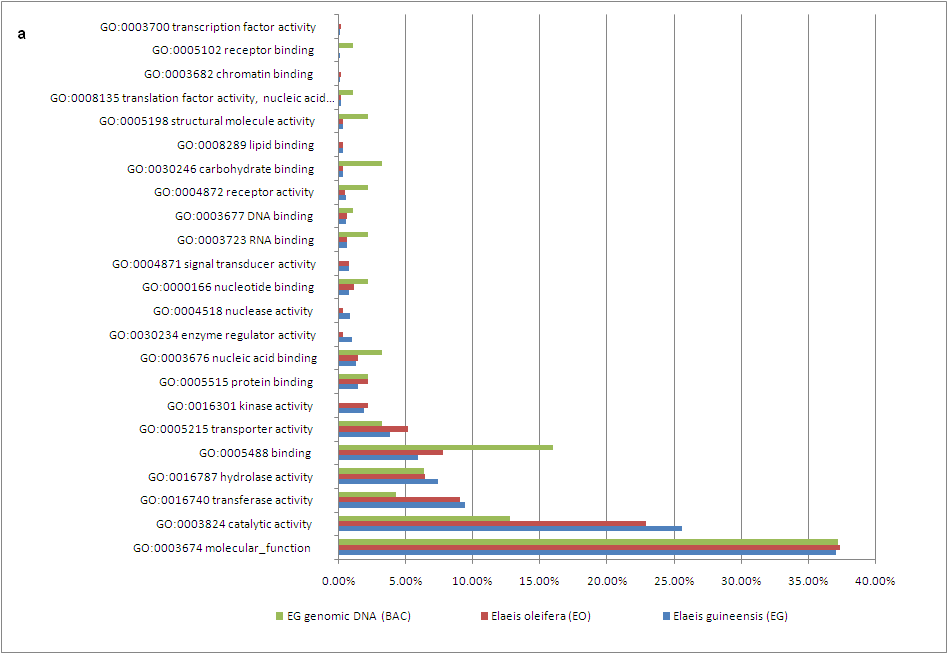
**

**B**

**
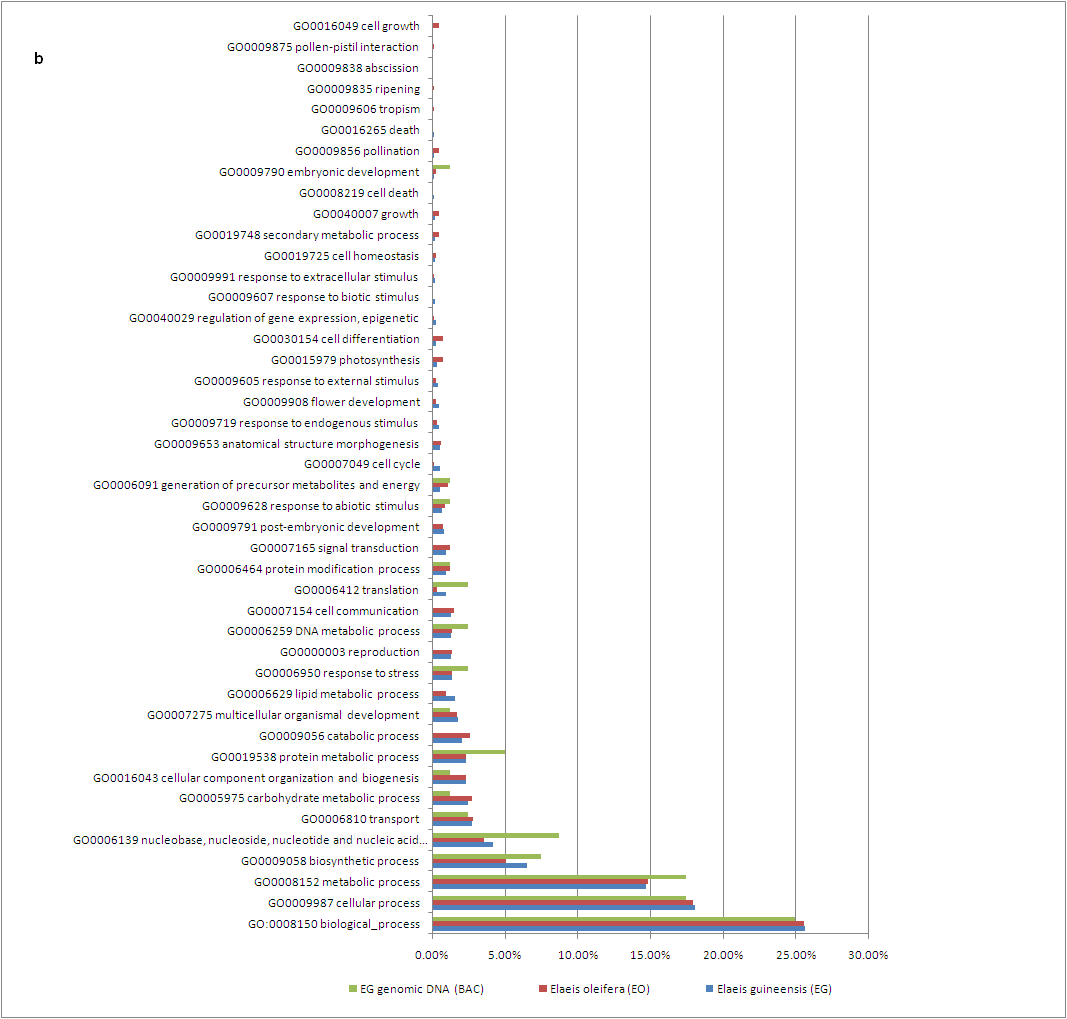
**

**C**

**
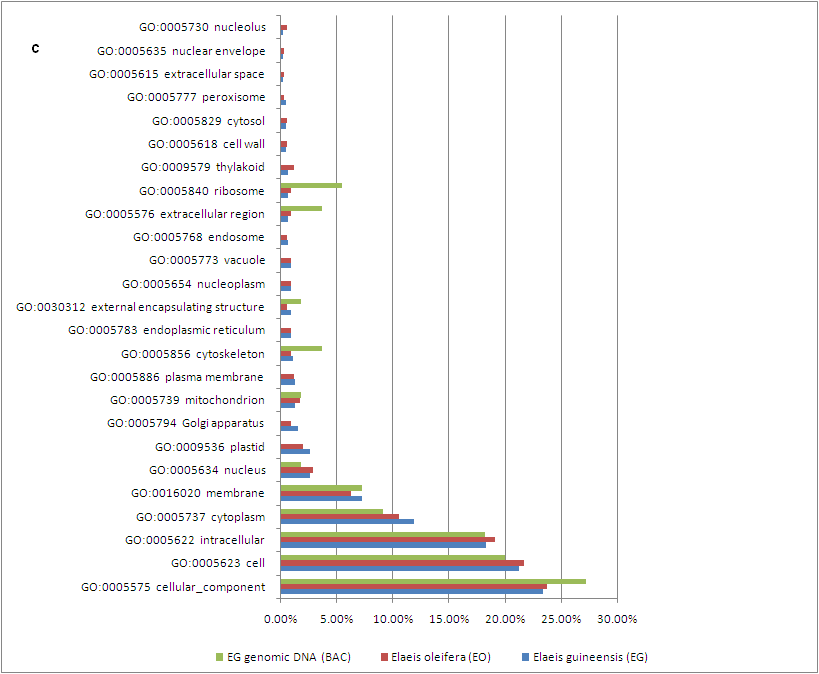
**

**Figure S1. Gene ontology classification of EG01, EO01 and BAC sequences. Three GO categories, [A] Molecular function (**ML**) [B] Biological process (BP), and [C] Cellular component (CC) terms were mapped to Plant Slim GO annotations using CateGOrizer.**
