# Supplementary material for: Analyses of Hypomethylated Oil Palm Gene Space
Source: PLoS One. 2014 Jan 30;9(1):e86728. doi: 10.1371/journal.pone.0086728 (PMC3907425; doi:10.1371/journal.pone.0086728)
Supplement: Figure S2 — Depth at SNP positions for [A] EG01 and [B] EO01 contigs. The red line indicates the cut-off of two standard deviation from mean, where the SNPs on the left of the line were defined as unreliable. (DOCX) [file pone.0086728.s002.docx]

**A**


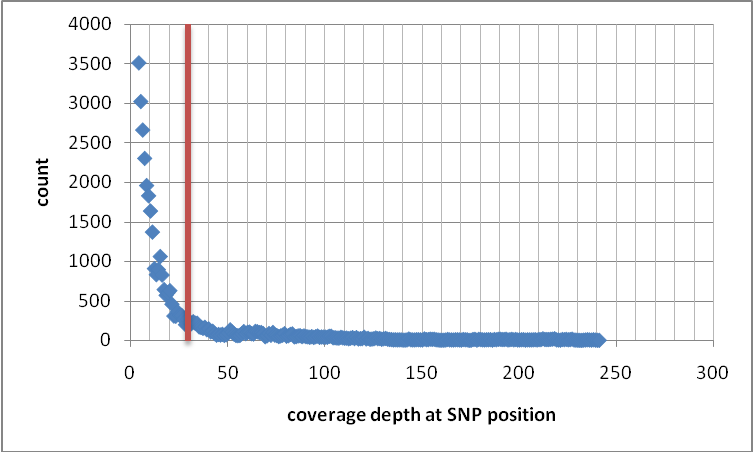


**B**


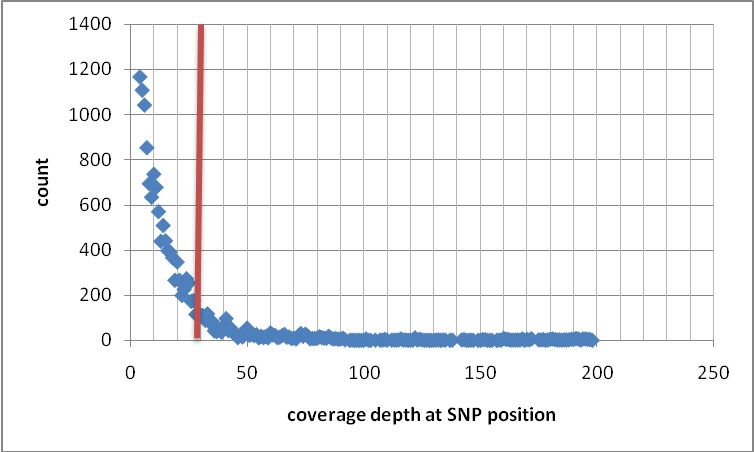


**Figure S2. Depth at SNP positions for [A] EG01 and [B] EO01 contigs.** The red line indicates the cut-off of two standard deviation from the mean, where the SNPs on the left of the line were defined as unreliable.
