# Supplementary material for: Analyses of Hypomethylated Oil Palm Gene Space
Source: PLoS One. 2014 Jan 30;9(1):e86728. doi: 10.1371/journal.pone.0086728 (PMC3907425; doi:10.1371/journal.pone.0086728)
Supplement: Table S1 — E. guineensis and E. oleifera filtered and unfiltered genomic library information. (DOCX) [file pone.0086728.s003.docx]

**Table S1. *E.* *guineensis* and *E.* *oleifera* filtered and unfiltered genomic library information**

| No | Species | | | Breeding Line | Genotype | Library | No. Sequences | | UF Library Pair ID | |
| --- | --- | --- | --- | --- | --- | --- | --- | --- | --- | --- |
| Methylation Filter (MF) | | | | | | | | | | |
| 1 | *E. guineensis* | | | Deli dura | Dura | 214 | 186 | | 213 | |
| 2 | *E. guineensis* | | | Deli dura | Dura | 233 | 17,253 | | 234 | |
| 3 | *E. guineensis* | | | Deli dura | Dura | 280 | 118,679 | | 514 | |
| 4 | *E. guineensis* | | | *AVROS* pisifera | Pisifera | 281 | 139,562 | | 512 | |
| 5 | *E. guineensis* | | | Deli dura x *AVROS* pisifera | D x P * (Tenera) | 301 | 5,798 | | 322 | |
| 6 | *E. guineensis* | | | Deli dura x *AVROS* pisifera | D x P * (Tenera) | 343 | 6,015 | | 361 | |
| 7 | *E. guineensis* | | | Deli dura x *AVROS* pisifera | D x P * (Tenera) | 321 | 6,622 | | 342 | |
| 8 | *E. oleifera* | | | Columbian oleifera | - | 282 | 144,932 | | 510 | |
| 9 | *E. oleifera* | | | Columbian oleifera | - | 302 | 5,812 | | 341 | |
|  |  | |  | |  | Total | 444,859 | |  | |
|  |  | |  | |  |  |  |  | |  |
| Unfiltered (UF) | |  |  |  |  |  |  |  |  |  |
| 10 | *E. guineensis* | | | Deli dura | Dura | 213 | 379 | |  |  |
| 11 | *E. guineensis* | | | Deli dura | Dura | 234 | 1,343 | |  |  |
| 12 | *E. guineensis* | | | Deli dura | Dura | 514 | 1,358 | |  |  |
| 13 | *E. guineensis* | | | *AVROS* pisifera | Pisifera | 512 | 2,877 | |  |  |
| 14 | *E. guineensis* | | | Deli dura x *AVROS* pisifera | D x P * (Tenera) | 322 | 1,093 | |  |  |
| 15 | *E. guineensis* | | | Deli dura x *AVROS* pisifera | D x P * (Tenera) | 361 | 2,661 | |  |  |
| 16 | *E. guineensis* | | | Deli dura x *AVROS* pisifera | D x P * (Tenera) | 342 | 2,732 | |  |  |
| 17 | *E. oleifera* | | | Columbian oleifera | - | 341 | 1,172 | |  |  |
| 18 | *E. oleifera* | | | Columbian oleifera | - | 510 | 2,812 | |  |  |
|  |  | | |  |  | Total | 16,427 | |  |  |

* D x P = Dura x Pisifera
