# Supplementary material for: Analyses of Hypomethylated Oil Palm Gene Space
Source: PLoS One. 2014 Jan 30;9(1):e86728. doi: 10.1371/journal.pone.0086728 (PMC3907425; doi:10.1371/journal.pone.0086728)
Supplement: Table S2 — Categorization of EG and EO genes into KEGG pathways. (DOCX) [file pone.0086728.s004.docx]

**Table S2. Categorization of EG and EO genes into KEGG pathways**

| **Pathways** | **EG** | **EO** |
| --- | --- | --- |
| ABC transporters | 1 | 0 |
| Alanine, aspartate and glutamate metabolism | 2 | 0 |
| alpha-Linolenic acid metabolism | 1 | 1 |
| Amino sugar and nucleotide sugar metabolism | 8 | 3 |
| Aminoacyl-tRNA biosynthesis | 6 | 0 |
| Ascorbate and aldarate metabolism | 2 | 1 |
| Basal transcription factors | 2 | 0 |
| Base excision repair | 3 | 1 |
| Biosynthesis of unsaturated fatty acids | 1 | 0 |
| Carotenoid biosynthesis | 4 | 1 |
| Circadian rhythm - plant | 3 | 0 |
| Citrate cycle (TCA cycle) | 8 | 2 |
| Cyanoamino acid metabolism | 3 | 0 |
| Cysteine and methionine metabolism | 10 | 5 |
| Diterpenoid biosynthesis | 4 | 1 |
| DNA replication | 3 | 1 |
| Endocytosis | 3 | 1 |
| Fatty acid biosynthesis | 9 | 0 |
| Fatty acid metabolism | 2 | 0 |
| Flavone and flavonol biosynthesis | 2 | 0 |
| Flavonoid biosynthesis | 1 | 2 |
| Folate biosynthesis | 0 | 1 |
| Fructose and mannose metabolism | 3 | 1 |
| Galactose metabolism | 1 | 0 |
| Glutathione metabolism | 1 | 0 |
| Glycerolipid metabolism | 5 | 1 |
| Glycerophospholipid metabolism | 3 | 1 |
| Glycine, serine and threonine metabolism | 8 | 2 |
| Glycolysis / Gluconeogenesis | 16 | 6 |
| Glycosaminoglycan degradation | 2 | 0 |
| Glycosylphosphatidylinositol(GPI)-anchor biosynthesis | 1 | 0 |
| Glyoxylate and dicarboxylate metabolism | 2 | 6 |
| Histidine metabolism | 1 | 1 |
| Inositol phosphate metabolism | 6 | 2 |
| Limonene and pinene degradation | 2 | 0 |
| Linoleic acid metabolism | 5 | 3 |
| Lipoic acid metabolism | 0 | 1 |
| Lysine biosynthesis | 1 | 0 |
| Metabolic pathways | 2 | 1 |
| N-Glycan biosynthesis | 4 | 2 |
| Nicotinate and nicotinamide metabolism | 1 | 0 |
| **Pathways** | **EG** | **EO** |
| Nitrogen metabolism | 1 | 1 |
| Non-homologous end-joining | 1 | 1 |
| Oxidative phosphorylation | 25 | 28 |
| Pantothenate and CoA biosynthesis | 1 | 0 |
| Pentose and glucuronate interconversions | 1 | 1 |
| Pentose phosphate pathway | 3 | 3 |
| Peroxisome | 0 | 1 |
| Phagosome | 6 | 1 |
| Phenylalanine metabolism | 9 | 2 |
| Phenylalanine, tyrosine and tryptophan biosynthesis | 2 | 1 |
| Phenylpropanoid biosynthesis | 3 | 2 |
| Photosynthesis | 8 | 15 |
| Photosynthesis - antenna proteins | 8 | 2 |
| Plant-pathogen interaction | 10 | 5 |
| Porphyrin and chlorophyll metabolism | 1 | 1 |
| Proteasome | 5 | 1 |
| Protein export | 1 | 2 |
| Protein processing in endoplasmic reticulum | 8 | 6 |
| Purine metabolism | 8 | 9 |
| Pyrimidine metabolism | 3 | 1 |
| Pyruvate metabolism | 1 | 1 |
| Ribosome | 23 | 8 |
| RNA degradation | 4 | 2 |
| SNARE interactions in vesicular transport | 0 | 1 |
| Spliceosome | 13 | 6 |
| Starch and sucrose metabolism | 13 | 3 |
| Steroid biosynthesis | 1 | 2 |
| Taurine and hypotaurine metabolism | 1 | 0 |
| Terpenoid backbone biosynthesis | 3 | 0 |
| Tryptophan metabolism | 1 | 3 |
| Tyrosine metabolism | 0 | 1 |
| Ubiquinone and other terpenoid-quinone biosynthesis | 4 | 1 |
| Ubiquitin mediated proteolysis | 4 | 1 |
| Valine, leucine and isoleucine biosynthesis | 1 | 1 |
| Zeatin biosynthesis | 2 | 1 |
