# Supplementary material for: Analyses of Hypomethylated Oil Palm Gene Space
Source: PLoS One. 2014 Jan 30;9(1):e86728. doi: 10.1371/journal.pone.0086728 (PMC3907425; doi:10.1371/journal.pone.0086728)
Supplement: Table S3 — Di-, tri- and tetranucleotide repeats identified in EG01. (DOCX) [file pone.0086728.s005.docx]

**Table S3. Di-, tri- and tetranucleotide repeats identified in EG01**

| SSR MOTIFS | TOTAL | Number of Repeats | | | | | | | | | | | | |
| --- | --- | --- | --- | --- | --- | --- | --- | --- | --- | --- | --- | --- | --- | --- |
|  |  | 4 | 5 | 6 | 7 | 8 | 9 | 10 | 11 | 12 | 13 | 14 | 15 | >16 |
| AAAC | 72 | 45 | 19 | 4 | 4 | - | - | - | - | - | - | - | - | - |
| AAAG | 533 | 319 | 117 | 55 | 15 | 10 | 8 | 7 | 1 | - | - | - | 1 | - |
| AAAT | 1121 | 630 | 295 | 113 | 51 | 16 | 5 | 6 | 1 | - | 1 | - | 1 | 2 |
| AAC | 195 | - | 98 | 40 | 29 | 15 | 5 | 1 | 2 | - | 2 | 1 | - | 2 |
| AACC | 34 | 28 | 3 | 2 | 1 | - | - | - | - | - | - | - | - | - |
| AACG | 1 | - | - | 1 | - | - | - | - | - | - | - | - | - | - |
| AACT | 5 | 4 | 1 | - | - | - | - | - | - | - | - | - | - | - |
| AAG | 1404 | - | 613 | 253 | 201 | 131 | 78 | 35 | 32 | 10 | 12 | 9 | 8 | 22 |
| AAGC | 10 | 9 | 1 | - | - | - | - | - | - | - | - | - | - | - |
| AAGG | 18 | 14 | 2 | 1 | - | 1 | - | - | - | - | - | - | - | - |
| AAT | 1385 | - | 495 | 260 | 186 | 124 | 64 | 44 | 29 | 21 | 24 | 21 | 9 | 108 |
| AATC | 30 | 27 | 2 | - | - | - | 1 | - | - | - | - | - | - | - |
| AATG | 20 | 16 | 3 | 1 | - | - | - | - | - | - | - | - | - | - |
| AATT | 232 | 60 | 148 | 16 | 3 | 3 | 2 | - | - | - | - | - | - | - |
| AC | 1933 | - | - | - | 436 | 334 | 279 | 200 | 150 | 119 | 92 | 73 | 48 | 202 |
| ACAG | 10 | 9 | 1 | - | - | - | - | - | - | - | - | - | - | - |
| ACAT | 831 | 336 | 167 | 93 | 47 | 53 | 31 | 29 | 18 | 9 | 18 | 4 | 4 | 22 |
| ACC | 264 | - | 154 | 52 | 29 | 12 | 8 | 4 | 3 | 1 | - | - | - | 1 |
| ACCC | 10 | 7 | 3 | - | - | - | 0 | - | - | - | - | - | - | - |
| ACCG | 91 | 59 | 10 | 6 | 3 | - | 1 | 1 | 1 | 2 | - | - | 1 | 7 |
| ACCT | 2 | 1 | 1 | - | - | - | - | - | - | - | - | - | - | - |
| ACG | 59 | - | 31 | 8 | 4 | 6 | 6 | 3 | 1 | - | - | - | - | - |
| ACGC | 48 | 27 | 14 | 4 | - | 1 | 2 | - | - | - | - | - | - | - |
| ACGG | 6 | 3 | 2 | - | 1 | - | - | - | - | - | - | - | - | - |
| ACGT | 11 | 6 | 2 | 2 | - | 1 | - | - | - | - | - | - | - | - |
| ACT | 38 | - | 20 | 11 | 1 | 3 | - | - | 2 | 1 | - | - | - | - |
| ACTC | 8 | 3 | 1 | 3 | - | 1 | - | - | - | - | - | - | - | - |
| ACTG | 1 | 1 | - | - | - | - | - | - | - | - | - | - | - | - |
| AG | 6761 | - | - | - | 1235 | 1001 | 854 | 681 | 570 | 419 | 352 | 330 | 255 | 1064 |
| AGAT | 116 | 65 | 22 | 13 | 8 | 2 | 2 | 1 | - | 2 | - | - | 1 | - |
| AGC | 303 | - | 138 | 74 | 39 | 26 | 9 | 5 | 1 | 5 | 4 | - | 2 | - |
| AGCC | 3 | 3 | - | - | - | - | - | - | - | - | - | - | - | - |
| AGCG | 45 | 28 | 16 | 1 | - | - | - | - | - | - | - | - | - | - |
| AGCT | 4 | 2 | 2 | - | - | - | - | - | - | - | - | - | - | - |
| AGG | 777 | - | 399 | 149 | 105 | 66 | 29 | 11 | 8 | 6 | - | 1 | - | 3 |
| AGGC | 6 | 5 | 1 | - | - | - | - | - | - | - | - | - | - | - |
| AGGG | 128 | 92 | 24 | 11 | 1 | - | - | - | - | - | - | - | - | - |
| AT | 6156 | - | - | - | 915 | 705 | 579 | 461 | 359 | 313 | 227 | 230 | 212 | 2155 |
| ATC | 255 | - | 143 | 55 | 23 | 12 | 10 | 8 | 1 | 2 | 1 | - | - | - |
| ATCC | 61 | 44 | 12 | 1 | 1 | 1 | 2 | - | - | - | - | - | - | - |
| ATCG | 11 | 5 | 2 | 3 | 1 | - | - | - | - | - | - | - | - | - |
| ATGC | 87 | 70 | 8 | 7 | 1 | 1 | - | - | - | - | - | - | - | - |
| CCCG | 3 | 2 | - | 1 | - | - | - | - | - | - | - | - | - | - |
| CCG | 471 | - | 217 | 110 | 66 | 31 | 30 | 11 | 2 | 1 | 1 | 2 | - | - |
| CCGG | 1 | 1 | - | - | - | - | - | - | - | - | - | - | - | - |
| CG | 61 | - | - | - | 41 | 8 | 7 | 4 | 1 | - | - | - | - | - |
| Total | 23,621 |  |  |  |  |  |  |  |  |  |  |  |  |  |
