# Supplementary material for: Analyses of Hypomethylated Oil Palm Gene Space
Source: PLoS One. 2014 Jan 30;9(1):e86728. doi: 10.1371/journal.pone.0086728 (PMC3907425; doi:10.1371/journal.pone.0086728)
Supplement: Table S4 — Di-, tri- and tetranucleotide repeats identified in EO01. (DOCX) [file pone.0086728.s006.docx]

**Table S4. Di-, tri- and tetranucleotide repeats identified in EO01**

| SSR MOTIFS | TOTAL | Number of repeats | | | | | | | | | | | | |
| --- | --- | --- | --- | --- | --- | --- | --- | --- | --- | --- | --- | --- | --- | --- |
|  |  | 4 | 5 | 6 | 7 | 8 | 9 | 10 | 11 | 12 | 13 | 14 | 15 | >16 |
| AAAC | 33 | 19 | 9 | 4 | - | 1 | - | - | - | - | - | - | - | 0 |
| AAAG | 293 | 172 | 70 | 23 | 13 | 5 | 7 | 2 | 1 | - | - | - | - | 0 |
| AAAT | 538 | 327 | 146 | 37 | 13 | 5 | 7 | 1 | 2 | - | - | - | - | 0 |
| AAC | 98 | - | 50 | 24 | 9 | 8 | 4 | 2 | - | - | - | - | - | 1 |
| AACC | 9 | 7 | - | 1 | 1 | - | - | - | - | - | - | - | - | 0 |
| AACG | 2 | 1 | - | 1 | - | - | - | - | - | - | - | - | - | 0 |
| AACT | 3 | 3 | - | - | - | - | - | - | - | - | - | - | - | 0 |
| AAG | 619 | - | 259 | 118 | 90 | 51 | 31 | 23 | 13 | 10 | 7 | 4 | 2 | 11 |
| AAGC | 4 | 4 | - | - | - | - | - | - | - | - | - | - | - | 0 |
| AAGG | 9 | 6 | 3 | - | - | - | - | - | - | - | - | - | - | 0 |
| AAGT | 1 | 1 | - | - | - | - | - | - | - | - | - | - | - | 0 |
| AAT | 597 | - | 231 | 136 | 76 | 34 | 29 | 17 | 13 | 11 | 7 | 8 | 4 | 31 |
| AATC | 11 | 8 | 2 | 1 | - | - | - | - | - | - | - | - | - | 0 |
| AATG | 11 | 8 | 3 | - | - | - | - | - | - | - | - | - | - | 0 |
| AATT | 73 | 20 | 47 | 6 | - | - | - | - | - | - | - | - | - | 0 |
| AC | 751 | - | - | - | 183 | 142 | 90 | 91 | 56 | 35 | 35 | 28 | 21 | 70 |
| ACAG | 4 | 1 | 3 | - | - | - | - | - | - | - | - | - | - | 0 |
| ACAT | 300 | 121 | 59 | 49 | 24 | 17 | 10 | 5 | 3 | 2 | 2 | 2 | 1 | 5 |
| ACC | 103 | - | 59 | 18 | 12 | 8 | 3 | 2 | 1 | - | - | - | - | 0 |
| ACCC | 7 | 7 | - | - | - | - | - | - | - | - | - | - | - | 0 |
| ACCG | 1 | 1 | - | - | - | - | - | - | - | - | - | - | - | 0 |
| ACCT | 3 | 2 | 1 | - | - | - | - | - | - | - | - | - | - | 0 |
| ACG | 28 | - | 11 | 7 | 3 | 1 | 2 | 1 | 2 | 1 | - | - | - | 0 |
| ACGC | 21 | 14 | 5 | 2 | - | - | - | - | - | - | - | - | - | 0 |
| ACGG | 1 | - | - | - | - | 1 | - | - | - | - | - | - | - | 0 |
| ACGT | 3 | 1 | 1 | - | - | 1 | - | - | - | - | - | - | - | 0 |
| ACT | 13 | - | 8 | 4 | 1 | - | - | - | - | - | - | - | - | 0 |
| ACTC | 2 | 2 | - | - | - | - | - | - | - | - | - | - | - | 0 |
| AG | 3004 | - | - | - | 567 | 454 | 380 | 271 | 254 | 177 | 184 | 132 | 131 | 454 |
| AGAT | 47 | 26 | 8 | 2 | 3 | 5 | 1 | - | 1 | 1 | - | - | - | 0 |
| AGC | 116 | - | 51 | 26 | 21 | 9 | 5 | 4 | - | - | - | - | - | 0 |
| AGCC | 3 | - | 2 | - | 1 | - | - | - | - | - | - | - | - | 0 |
| AGCG | 15 | 8 | 5 | 2 | - | - | - | - | - | - | - | - | - | 0 |
| AGG | 339 | - | 164 | 65 | 49 | 34 | 14 | 10 | 2 | - | 1 | - | - | 0 |
| AGGC | 2 | 2 | - | - | - | - | - | - | - | - | - | - | - | 0 |
| AGGG | 52 | 30 | 15 | 5 | 2 | - | - | - | - | - | - | - | - | 0 |
| AT | 2591 | - | - | - | 419 | 325 | 241 | 200 | 171 | 117 | 106 | 106 | 84 | 822 |
| ATC | 125 | - | 70 | 23 | 13 | 6 | 6 | 3 | 1 | - | - | 1 | 1 | 1 |
| ATCC | 27 | 19 | 5 | 2 | 1 | - | - | - | - | - | - | - | - | 0 |
| ATCG | 6 | 5 | - | - | 1 | - | - | - | - | - | - | - | - | 0 |
| ATGC | 35 | 31 | 4 | - | - | - | - | - | - | - | - | - | - | 0 |
| CCCG | 2 | - | - | 1 | 1 | - | - | - | - | - | - | - | - | 0 |
| CCG | 209 | - | 88 | 53 | 33 | 12 | 11 | 7 | 3 | - | 1 | - | - | 1 |
| CG | 20 | - | - | - | 16 | 2 | - | 1 | 1 | - | - | - | - | 0 |
| Total | 10,131 |  |  |  |  |  |  |  |  |  |  |  |  |  |
