# Supplementary material for: Analyses of Hypomethylated Oil Palm Gene Space
Source: PLoS One. 2014 Jan 30;9(1):e86728. doi: 10.1371/journal.pone.0086728 (PMC3907425; doi:10.1371/journal.pone.0086728)
Supplement: Table S5 — List of perfect and partial match miRNAs from EG01 and EO01 contigs. (DOCX) [file pone.0086728.s007.docx]

**Table S5. List of perfect and partial match miRNAs from EG01 and EO01 contigs**

| **Oil Palm Contigs** | **Start Position** | **End Position** | **Best Hits with miRNAs in miRBase** | **Match Status** |
| --- | --- | --- | --- | --- |
| EGC01043189 | 69 | 149 | peu-MIR2911 | Perfect |
| EGC01002494 | 1402 | 1482 | peu-MIR2911 | Perfect |
| EGC01007640 | 480 | 560 | peu-MIR2911 | Perfect |
| EGC01002621 | 727 | 817 | peu-MIR2916 | Perfect |
| EGC01009851 | 1007 | 1097 | peu-MIR2916 | Perfect |
| EGC01006056 | 1193 | 1273 | peu-MIR2911 | Perfect |
| EGC01000570 | 181 | 243 | peu-MIR2914 | Perfect |
| EGC01004418 | 1477 | 1539 | peu-MIR2914 | Perfect |
| EGC01005984 | 1347 | 1423 | peu-MIR2911 | Perfect |
| EGC01029522 | 271 | 361 | ptc-MIR156j | Perfect |
| EOC01000015 | 962 | 1052 | peu-MIR2916 | Perfect |
|  | 283 | 345 | peu-MIR2914 |  |
|  | 1266 | 1324 | peu-MIR2910 |  |
| EOC01013908 | 83 | 145 | peu-MIR2914 | Perfect |
| EOC01002900 | 1203 | 1286 | vvi-MIR156i | Perfect |
| EOC01002804 | 920 | 998 | pta-MIR1310 | Perfect |
| EOC01008865 | 68 | 241 | vvi-MIR319f | Perfect |
| EOC01001645 | 798 | 910 | sbi-MIR167g | Perfect |
| EOC01006693 | 943 | 1044 | ptc-MIR319e | Perfect |
| EOC01010601 | 6 | 380 | vvi-MIR845a | Perfect |
| EOC01007557 | 285 | 619 | vvi-MIR845b | Perfect |
| EGC01005329 | 228 | 306 | pta-MIR1310 | Partial |
| EGC01020272 | 50 | 133 | osa-MIR160b | Partial |
| EGC01029613 | 1 | 145 | vvi-MIR159c | Partial |
| EGC01016260 | 945 | 1117 | vvi-MIR319f | Partial |
| EGC01034556 | 314 | 424 | bdi-MIR156 | Partial |

| **Oil Palm Contigs** | **Start Position** | **End Position** | **Best Hits with miRNAs in miRBase** | **Match Status** |
| --- | --- | --- | --- | --- |
| EGC01009392 | 1088 | 1177 | gma-MIR390a | Partial |
| EGC01025980 | 958 | 1000 | mtr-MIR169a | Partial |
| EGC01019990 | 761 | 823 | vvi-MIR171g | Partial |
| EGC01004561 | 980 | 1087 | sbi-MIR171f | Partial |
| EGC01040072 | 611 | 652 | ptc-MIR156i | Partial |
| EGC01009284 | 1081 | 1216 | osa-MIR166g | Partial |
| EGC01013647 | 380 | 468 | vvi-MIR171b | Partial |
| EGC01028315 | 657 | 780 | ptc-MIR168b | Partial |
| EGC01038441 | 146 | 239 | ptc-MIR395h | Partial |
| EGC01008132 | 606 | 669 | ptc-MIR171h | Partial |
| EGC01037880 | 303 | 491 | gma-MIR1536 | Partial |
| EGC01027039 | 1 | 253 | vvi-MIR845a | Partial |
| EGC01024333 | 431 | 806 | vvi-MIR845a | Partial |
| EOC01018020 | 111 | 149 | cre-MIR916 | Partial |
| EOC01007681 | 130 | 178 | cre-MIR916 | Partial |
| EOC01003978 | 1011 | 1059 | cre-MIR916 | Partial |
