# Supplementary material for: Analyses of Hypomethylated Oil Palm Gene Space
Source: PLoS One. 2014 Jan 30;9(1):e86728. doi: 10.1371/journal.pone.0086728 (PMC3907425; doi:10.1371/journal.pone.0086728)
Supplement: Materials S1 — Formula to calculate gene enrichment. (DOCX) [file pone.0086728.s012.docx]

SUPPLEMENTARY MATERIALS 1

**Gene Enrichment Method**

The gene enrichment filter power calculation for *E. guineensis* and *E. oleifera* as described in Bedell et al., (2005) is:

p^mf^

g

_______

p^μf^

g

p^mf^ is the probability that a methylation filtered (MF) read sample gene coding sequence

g

p^μf^ is the probability that an unfiltered (UF) read sample gene coding sequence

g

These probabilities are estimated by looking at the proportions of MF and UF reads that hit genes

^

p^mf^ = N^mf^

g

g

___________

N^mf^

^

p^μf^ = N^μf^

g

g

___________

N^μf^

It was observed that out of N^mf^ methylation filtered reads, N^mf^ of them hit a gene and for N^μf^ unfiltered reads, N^μf^ of them hit genes. The 95% confidence intervals for these binomial proportions are calculated using the standard approximate formula, *viz*.:

g

g

^

^

^

p^μf^ ± 1.96 p^μf^ (1 - p^μf^ )

g

g

g


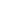


___________________________

N^μf^

For the palm data analysis, we used the mixed UF and MF assembly to sample in depth the filtered and unfiltered reads.

N^mf^ = number of filtered reads in contigs (excluding singletons)

N^μf^ = number of unfiltered reads in contigs (excluding singletons)

N^mf^ = number of filtered reads in (repeat-masked) contigs with a hit to a gene

g

N^μf^ = number of filtered reads in (repeat-masked) contigs with a hit to a gene

g
