# Supplementary material for: Analyses of Hypomethylated Oil Palm Gene Space
Source: PLoS One. 2014 Jan 30;9(1):e86728. doi: 10.1371/journal.pone.0086728 (PMC3907425; doi:10.1371/journal.pone.0086728)
Supplement: Materials S2 — Formula to calculate genome sampling. (DOCX) [file pone.0086728.s013.docx]

SUPPLEMENTARY MATERIALS 2

**Genome Sampling Method**

Modifications were made for our palm assemblies because the MF and UF reads were assembled together.

The Lander Waterman formula for the expected number of islands in an assembly of *N* reads of length *L* (bp) randomly sampled from a genome of size *G* (bp), with at least *T* (bp) required overlap is:

*Ne^-cσ^*

Where,

*LN* *N*

*c* *= ----- = Lp (p* = ---- probability a read starts at a given site)

*G* *G*

*L* – *T*

*σ = -------*

*L*

Definitions

On average, a clone generates k non-overlapping reads, where 1≤ *k* ≤ 2. Since *k* only counts overlapping pairs as single sequence events, a process of collapsing paired-end reads into paired-end-contigs was done so as to adjust the calculation of k using the resulting number of distinct contigs and un-collapsed singletons (Whitelaw et al., 2003).

Description:

G_f_ = effective size of filtered genome to be estimated from the number of islands

G_g_ = size of complete genome

N_f_ = number of clones sampled from G_f_

N_g_ = number of clones sampled from G_g_

*N_f_*

*p_f_ = -------* is the probability of starting an MF clone at a given base pair of the reduced genome.

*G_f_*

The probability of starting an MF clone from either of its end reads at a given genome position is approximately *kp****_f_***.

*N_g_*

*p_g_ = -------* is the probability of starting an UF clone at a given base pair of the reduced genome.

*G_g_*

The probability of starting a UF clone from either of its end reads at a given genome position is approximately *kp_g_*.

The probability of sampling either an MF or UF clone at a given base in the reduced genome is:

~

*p_g_* + *p_f_* – *p_g_ . p_f_*  = *p_g_* + *p_f_*

**MF tagged islands**

The probability of either paired-end read of an MF clone starting at a position in the genome but not overlapping with either of the paired end reads of any other UF or MF clone is:

*kp_f_* (1 – *kp_f_* – *kp_g_*) ^(^*^L^*^-^*^T^*^)^

*^kp^_f_^+kp^_g_ ^L-T^*

= *kp_f_* (1 – *kp_f_* – *kp_g_*)*^kp^_f_^+kp^_g_ ^L^*

*^L^*

*c_f +_ c_g_*

*σ*

= *kp_f_* (1 – *kp_f_* – *kp_g_*) *^kp^_f_^+kp^_g_*

~

= *kp_f_ e^-(c^f ^+cg)σ^*

where,

*kLN_f_*

*c_f_ = -------- = kLp_f_*

*G_f_*

*kLN_g_*

*c_g_ = -------- = kLp_g_*

*G_g_*

*L - T*

*σ = --------*

*L*

Hence, the expected number of MF-tagged islands is:

*I_f_* = *kG_f_p_f_ e^-(c^f ^+cg)σ^*

= *kN_f_ e^-(c^f ^+cg)σ^*

From this and the known approximated size of the complete genome, the estimated effective size of the reduced genome is:

*-kLN_f_ σ*

*G_f_ = --------------------*

*I_f_*

*ln ----- + c_g_ σ*

*kN_f_*

**UF tagged islands**

A similar argument gives:

*I_g_* = *kN_g_ e^-(c^f ^+cg)σ^*

**All islands**

Thus, the total number of islands expected for a mixed assembly of UF and MF is:

*I_t_ = I_g_ + I_f_ = k(N_g_ + N_f_) e^-(c^f ^+cg)σ^*

Which provides an estimate of the approximate size of the filtered genome, using the combined counts of all the islands:

*-kLN_f_ σ*

*G_f_ = -----------------------------*

*I_t_*

*ln ---------------- + c_g_ σ*

*k(N_g_ +N_f_)*

This is essentially the formula of Whitelaw et al. (2003) with an additional term (*c_g_ σ*) in the denominator to adjust for the mixed assembly. Whitelaw’s formula is:

*-N(L – T)*

*G_f_ = -------------------*

*N_island_*

*ln---------*

*N*

The corresponding terms being :

In the denominator -

*I_t_ N_island_*

*ln ---------------- ≡ ln ---------*

*k(N_g_ +N_f_) N*

In the numerator –

*kNf ≡ N*

*L - T*

*Lσ ≡ L -------- = L - T*

*L*
